# Digital economy, technological progress, and city export trade

**DOI:** 10.1371/journal.pone.0269314

**Published:** 2022-06-03

**Authors:** Linlin Zhang, An Pan, Shuangshuang Feng, Yaoyao Qin

**Affiliations:** 1 School of Economics, Zhongnan University of Economics and Law, Wuhan, Hubei, China; 2 School of Finance & Economics, Wuhan College, Wuhan, Hubei, China; Universidad Nacional Autonoma de Nicaragua Leon, NICARAGUA

## Abstract

The development of the digital economy is conducive to the innovative development of foreign trade and the formation of a “dual circulation” development pattern in China. Based on the panel data of 285 prefecture-level cities in China from 2005 to 2019, this paper examines the influence of the digital economy on urban export trade and its heterogeneity. And we use a mediating effect model to explore the possible mediating role of technological progress in the above influences. The results find that: (1) The improvement of the digital economy can promote cities export; (2) The promotion of the digital economy to the growth of city export scale is heterogeneous, which is more significant in the western and northeastern cities with relatively remote geographical locations, and the third-tier and lower cities with relatively backward economic development. (3) Technological progress has played a significant role in promoting the growth of export for the digital economy. Thus, it’s of great importance for China to increase investment in digital economy infrastructure and pay more attention to the differences in diverse city development processes. It should also support basic research and development in information technology to promote high-quality development of China’s foreign trade through the digital economy.

## Introduction

Since the reform and opening-up, China’s foreign trade has achieved a leapfrog increase. Export has expanded from 16.76 billion yuan in 1978 to 21.73 billion yuan in 2021, with a 17.7% average annual growth. However, the early development of China’s foreign trade relied on its comparative advantage of low factor cost. Achieving innovative and sustainable development of trade is a severe problem China faces. During the 13th Five-Year Plan (2016–2020) period, China valued the digital economy and repeatedly mentioned building a “digital China”. Moreover, China has issued a series of relevant policy documents and measures to help construct the digital economy. The 14th Five-Year Plan (2021–2025) also emphasized it again. The General Office of the State Council laid out a policy “to promote the high-quality and innovative development of the foreign trade sector”, which added new impetus to the sector with innovative business models and offered new incentives to grow cross-border e-commerce. Digital trade has been another growth engine for foreign trade in China under the motivation of relevant measures.

Meanwhile, China’s digital trade has increased from 11 trillion yuan in the early phase of the 13th Five-Year Plan to 39.2 trillion yuan in 2020. According to China Internet Network Information Center (CNNIC), China’s Internet penetration rate will reach 71.6% by June 2021. The new development paradigm featuring dual circulation, in which domestic and overseas markets reinforce each other, with the domestic market as the mainstay, has brought a further boost in consumption to the Internet economy. Consumption has been upgraded both in terms of quality and quantity. The new online shopping models and the e-commerce industry have generated the domestic market, and cross-border e-commerce has energized the domestic and overseas markets. Therefore, the digital economy has become solid support to the innovative development of China’s foreign trade, especially the export trade.

Advancing the wave of digital technologies continuously, more and more scholars focus on the influence of the digital economy development on export. Most analyses from the national, industry, and enterprise levels believe that digital technologies reduce the trade cost and break the limitation of traditional transaction time and trading venues to increase the export. At the national level, some scholars support that digital technology promotes export due to overcoming geographic restrictions [1–3]. Moreover, Kurihara and Fukushima found that digitalization played a more prominent role in promoting export in developing countries than in developed countries in Asia [4]. At the industry level, Elia et al. based in the B2C industry (Design and Furniture, Fashion, and Food and Beverage), believe that digitalization boosts exports, with the fashion and apparel industry more benefiting from digital technology [5]. Subsequently, scholars have turned analysis from the macro-level to the micro with the gradual enrichment of micro-databases. Denicolai et al. found that digital technologies drive exports and that firms of different sizes and industries have different propensities to engage in digital exports [6]. The above researches have verified the digital economy promotion effect on the export from different ways, but also found it has a heterogeneity effect, such as differing with economic development, industrial characteristics, and types of firms.

Furthermore, the digital economy puts forward higher demand for technological progress based on the broad integration of digitalization and economic development. On the one hand, digitization acts as a “lubricant” to accelerate the flow of factor resources and boost technological progress by promoting capital accumulation and R&D capabilities [7–9]. In addition, it supports industrial integration and leads to more “technology spillover effects” through the extension of the trade chain [10]. On the other hand, concerning the relationship between technological progress and export trade, most literature studies the technical spillover effect of exports and believes that exports promote technological progress. Still, some literature also finds that technological progress can increase production and operation efficiency, thereby enhancing trade competitiveness and promoting export growth [11, 12]. Then, combining the above two aspects, can it be considered that the digital economy ultimately impacts export trade through the intermediary role of technological progress?

To sum up, the existing research has conducted various discussions on the influence of the digital economy on export trade. It has shown the following characteristics: First, more literature analyzed from the national and industrial levels, and cities are rarely taken as the research object, so they cannot reveal the possible heterogeneous influence. Second, there are few kinds of literature discussing the potential mediating role in the influence of the digital economy on export trade, so it is difficult to reveal the influence mechanism among them fully. In order to make up for the lack of available literature, this paper attempts to extend the existing research from the following aspects: Firstly, we find that there is a facilitating effect of the digital economy on city export trade, and that the effect is heterogeneous according to the cities’ geographical location and comprehensive economic strength; Secondly, technological progress is measured in terms of green total factor productivity, which reveals its significant mediating role in the impact of the digital economy on export trade.

## Theoretical analysis and research hypothesis

With the wide application of digital technologies such as big data, cloud computing, and the Internet of Things in social life, production, circulation, and other fields, the influence of the digital economy on export trade has attracted the attention of many scholars. Empirical tests have been carried out on the effect of the digital economy from different aspects. Accordingly, this paper combines the analysis ideas of existing relevant research, mainly from two parts of direct and indirect effects, carries out theoretical analysis on how the digital economy affects urban export trade, and puts forward corresponding research hypotheses.

### Direct effect of the digital economy on city export trade

The emergence and rapid development of the digital economy profoundly change the way, object, and content of trade. Digital technology can crucially reduce trade costs and expand trade markets across distance and time, thereby contributing to export growth. To this end, we can analyze the direct effect of the digital economy on export trade from the following two dimensions: First, the digital economy can reduce the cost of city export trade. New Economic Geography believes that trade cost is the key to trade location selection, spatial agglomeration, and diffusion of economic activities [13, 14], while export growth mainly comes from the reduction of cross-border trade cost [15–17]. Generally speaking, trade costs mainly include transaction costs, search costs, communication costs, and transportation costs [18]. The development of the digital economy will help reduce the above trade costs. Specifically, the transaction process between manufacturers and consumers in the digital economy is more convenient and efficient. The “disintermediation” effect gradually avoids a series of costs generated through intermediary transactions. With the vast popularity and application of digitalization, information on both sides of the business can be searched through the web, thus reducing the search costs. That needs to deliver via mail or telegrams of content, can real-time communication through the internet, thereby reducing the communication cost. Verhoef et al. believe that digital technology in blockchain and artificial intelligence can reduce logistics costs [19].

Second, the digital economy has expanded trade markets. Modern information and communication technologies have become an essential factor in economic behavior. Based on the development of the digital economy, the participants and business scope of the export trade market have broken the original geographical restrictions [1, 20]. In terms of participants, the development of the digital economy has unified the real economy and fictitious economy, and more and more virtual enterprises which have broken through geographical restrictions and improved the efficiency of trade have emerged. In terms of business scope, advances in information technology have enriched financial and educational products and weakened the restrictions on trade in services in different locations. Service products realize digitalization and tradability [14], thus promoting business development. Based on the theoretical analysis of the above two dimensions, this paper proposes the following research hypothesis:

Hypothesis 1: Digital economy can promote the growth of the export scale.

Further, the role of the digital economy in promoting city export trade may vary according to the stage of regional development. Unbalanced development theory holds that economic growth is not synchronous in space and time, and the economy develops inevitably with the transfer of various factors of production from peripheral areas to central areas. The story of the digital economy depends to a large extent on the investment of R&D, the capacity of skilled labor, and the level of digital infrastructure construction in the region [21, 22], such as the number of fiber-optic broadband users, network coverage, IPv6 address number and so on [23]. However, due to different stages of economic development between cities, all kinds of production factors in regional aggregation degrees are different, so the digital economy development shows the characteristics of uneven space [24]. A leading city, which will be more attractive to production factors such as technology, capital, and workforce, is conducive to the construction and improvement of digital economy infrastructure and holds more competitive advantages in foreign export trade [25].

At the same time, some scholars have found that the digital economy follows the law of diminishing marginal effect in terms of efficiency gains and value creation [26]. For regions with a higher level of economic development, more investment in digital construction has a diminishing marginal contribution effect on export trade [27]. Conversely, the marginal contribution of the digital economy to export trade is more remarkable for less developed cities. Where the lack of production factors leads to the slow development of the digital economy, the economies of scale and scope generated by minor improvements in infrastructure are more pronounced [28]. Therefore, there will be heterogeneity in the promotion effect of the digital economy on different cities’ exports. Based on this, this paper proposes the following research hypothesis:

Hypothesis 2: For cities at different stages of economic development, the effect of the digital economy on export is heterogeneous.

### Indirect effect of the digital economy on city export trade

According to the available studies, the digital economy effectively enhances technological innovation ability, promoting the development of city export trade. Information in the market is usually asymmetric, affecting the efficiency of the market allocation of resources. Digitalization speeds up the flow of market information, reduces the cost of information transmission, and thus improves technology and production efficiency. According to the conclusions of Antonopoulos et al. [29], Lyytinen et al. [30], and Meltzer [31], the digital economy plays a significant role in both short-term and long-term in improving production, management, and operation efficiency. It is an important reason for driving technological progress and efficiency.

On the other hand, technological progress is conducive to the growth of city exports. Firstly, as an endogenous driving force of city economic development [32], technological progress can encourage more economic subjects to participate in international trade and competition by improving production efficiency and innovation ability, thus promoting export trade development. Secondly, trade liberalization reallocates production factors between low-tech and high-tech economic agents [33]. Because of the entry barriers in foreign markets, there is a “self-selection” effect in export, with only high-skilled and high-productivity economic agents choosing to export [5, 34–37]. Accordingly, we propose the following research hypothesis:

Hypothesis 3: Technological progress plays an intermediary role in promoting the growth of city export by the digital economy.

**Fig 1:**
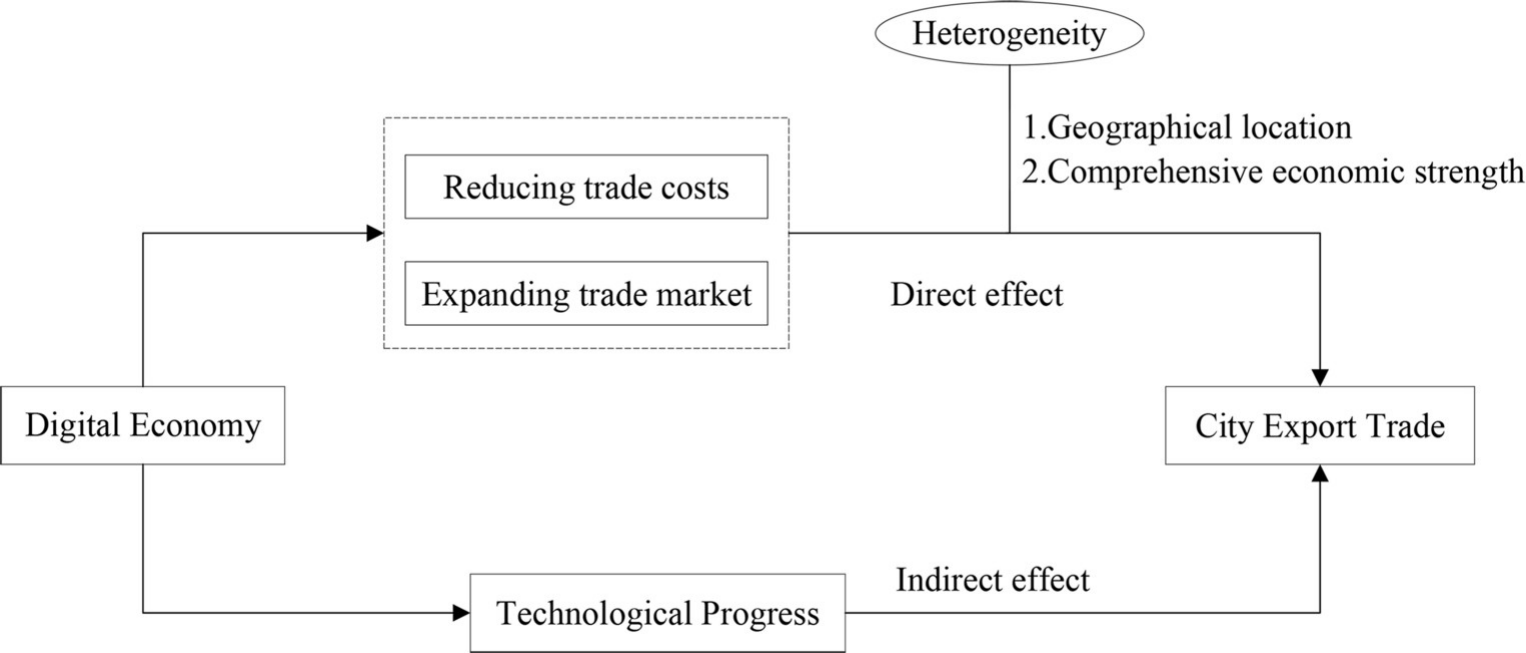
Pathways for the influence of the digital economy on city export trade.

To sum up, the effects of the digital economy on city export trade can be divided into direct effects and indirect effects through technological progress. The specific pathways of influence can be seen in Fig 1.

## Model constructing

The following benchmark model is constructed to examine the influence of the digital economy on city export trade.


$${\ln EX}_{it}=\delta_{0}+\delta_{1}{\ln Digit}_{it}+\delta_{2}X_{it}+\mu_{i}+\lambda_{t}+\varepsilon_{it}$$ (1)

Where subscript *i* represents different cities, *t* represents time; *EX* is the export scale of the city, *Digit* is the city’s digital economy development level, and *X* is other control variables. *μ* represents the individual effect of the city unaffected by the time factor, *λ* represents the time factor unaffected by the individual characteristics of the city, and *ε* is the random disturbance term. To reduce the possible influence of heteroscedasticity, we perform logarithmic processing on relevant variables; that is, it takes the natural logarithm for ln.

To test whether there is a mediating role of technological progress in the digital economy affecting city export trade, this paper further takes technological progress as a mediating variable based on Eq (1) and constructs the following mediating effect model.


$${TFP}_{it}=\alpha_{0}+\alpha_{1}{\ln Digit}_{it}+\alpha_{2}X_{it}+\mu_{i}+\lambda_{t}+\varepsilon_{it}$$ (2)

$${\ln EX}_{it}=\beta_{0}+\beta_{1}{\ln Digit}_{it}+\beta_{2}{TFP}_{it}+\beta_{3}X_{it}+\mu_{i}+\lambda_{t}{}_{}+\varepsilon_{it}$$ (3)

In Eqs (2) and (3), TFP is the mediating variable, which refers to technological progress, and other variables have the same meaning as Eq (1). As for whether there is a significant mediating effect, this paper will refer to the test method of Baron and David without elaborating [38].

## Variable selection and sources of data

### Variable selection

#### Explained variable

The export volume of each city measures export size (*EX*).

#### Core explanatory variable

A digital economy composite score based on five dimensions measures digital economy (*Digit*). Specifically, based on the digital economy index compiled by Katz and Koutroumpis [7], Hagsten and Kotnik [39], and the China Academy of Information and Communications Technology (2021), we select five dimensions to measure the digital economy, including total capita telecom business volume, total capita postal business volume, the number of employees in the information industry, the number of internet users and the number of mobile phone users at the end of the year. Then we use the entropy weight method to get a comprehensive score of the digital economy, which measures the development level of the digital economy in cities [40].

#### Mediating variable

Technological progress (*TFP*): It is measured by green total factor productivity. We construct the GML (Global Malmquist-Luenberger) index to comprehensively reflect prefecture-level cities’ technological progress [41–43]. Considering the availability of data, the factors input index and output index selected in this paper include: (1) Input: Capital input, taking the total investment in fixed assets of the whole society as capital input; Energy input, it is decomposed by the total electricity consumption of each city; Labor input, which is measured by the number of employees at the end of each year [44, 45]. (2) Good output: using the GDP of each city as good output. (3) Bad output: the emissions of industrial sulfur dioxide, industrial wastewater, and industrial soot in prefecture-level cities were selected as bad output.

#### Control variables

Generally speaking, regions with developed export trade are more likely to be at a higher level of economic development and openness, have a reasonable industrial structure, and use more advanced technology and skilled labor. Therefore, we add five control variables to our model. Degree of economic development (*grp*): It’s measured by per capita GDP in accordance with the usual practice. Degree of openness (*fdi*): Since FDI can bring advanced technology and management experience to the host country through spillover effect to improve the quality and quantity of export trade, this paper takes the ratio of actual utilized FDI to GDP to represent the degree of openness. Industrial structure (*is*): The percentage of the added value of the tertiary industry to GDP is used to describe the industrial structure. R&D (*rd*): The number of scientific research and technology employees measures it. Human capital(*hr*): This paper measures by the number of employees in the education industry.

### Sources of data and descriptive statistics

Considering data availability, this paper selects the data of 285 prefecture-level cities in China from 2005 to 2019 as samples for empirical analysis. Relevant data are mainly from China City Statistical Yearbook, China Statistical Yearbook, China Statistical Yearbook for Statistical Economic, and China Energy Statistical Yearbook. Table 1 shows descriptive statistics of variables.

**Table 1:** Summary statistics for variables.

Variable	Observations	Mean	Sd	Min	Max
ln*EX*	4275	13.064	2.196	2.031	19.043
*Digit*	4275	0.045	0.079	0.000	0.875
ln*TFP*	4275	0.963	0.207	0.132	2.031
*fdi*	4275	1.850	1.962	0.000	20.518
*is*	4275	38.969	9.660	8.580	83.520
*grp*	4275	0.367	0.188	0.030	1.980
ln*hr*	4275	1.672	0.561	0.086	4.070
ln*rd*	4275	0.490	0.545	0.010	4.287

## Results for benchmark model and its robust test

### Benchmark regression results

According to the F test and Hausman test, the two-way fixed-effect model was used in this paper for regression estimation of Eq (1). The estimated results in Table 2 were all robust standard error calculations adjusted by Driscoll and Kraay to reduce the influence of heteroscedasticity on results [46]. Based on the estimation results in column (6), it can be seen that the estimated coefficient of the variable *Digit* is significantly positive after controlling for the relevant variables, indicating that the digital economy has a significant contribution to the export, a result confirming the Hypothesis 1.

**Table 2:** Results of the benchmark model.

Variable	(1)	(2)	(3)	(4)	(5)	(6)
*Digit*	1.616***	1.597***	1.678***	1.839***	1.476***	1.405***
	(2.685)	(2.789)	(2.932)	(3.097)	(2.807)	(2.817)
*fdi*		0.038***	0.034**	0.031***	0.035***	0.036***
		(3.951)	(2.569)	(2.782)	(3.136)	(3.017)
*is*			-0.026***	-0.023***	-0.022***	-0.022***
			(-4.452)	(-3.605)	(-3.007)	(-3.008)
*grp*				0.907***	1.017***	1.033***
				(2.886)	(3.030)	(3.028)
ln*hr*					0.698***	0.638***
					(3.871)	(3.107)
ln*rd*						0.091
						(0.819)
Constant	12.059***	11.974***	12.935***	12.666***	11.497***	11.559***
	(469.553)	(371.387)	(51.771)	(40.946)	(21.125)	(22.196)
City effect	Yes	Yes	Yes	Yes	Yes	Yes
Year effect	Yes	Yes	Yes	Yes	Yes	Yes
Observations	4275	4275	4275	4275	4275	4275

Notes: The t-statistic in parenthesis.

***, **, and * indicate statistically significant at 1%, 5%, and 10%, respectively.

For control variables, the coefficient of *fdi* on export is significantly positive because FDI can influence export through scale effect, structural effect, and technological effect. The coefficient of *is* on export is highly negative, indicating that the excessive proportion of the tertiary industry is not conducive to export; that is, the internal structure of the tertiary sector still needs to be further improved. And the excessive pursuit of tertiary industrial scale and neglect of the rationality of the structure will lead to a certain degree of “industrial low-end”. The coefficient of *grp* on exports is significantly positive, which may be due to the improvement of the regional economic development drives the optimization and upgrading of the export trade structure, thereby promoting regional exports. The coefficient of ln*hr* indicates human capital can promote the efficiency of labor productivity in industries by providing a highly qualified workforce and optimal matching of workforce and jobs, thus increasing the domestic exports. the proportion of higher production factors in society. For the variable ln*rd*, the coefficient is positive but not significant. It suggests that R&D capabilities can enhance export competitiveness by increasing one’s innovation, but this may be due to the mismatch of resources invested in R&D in different disciplines, resulting in less investment in basic R&D and a lower input-output ratio, which in turn leads to R&D not playing a significant role.

### Robustness of benchmark model

To test the robustness of the estimation results of the benchmark model, this paper further conducted robustness tests from three aspects: considering endogeneity, replacing core explanatory variables, and eliminating outliers. The results are shown in Table 3. Firstly, considering that there may be bi-directional causality between digital economy and export trade, we take the first-order lag of variable *Digit* as an instrumental variable and use the two-stage least square method for estimation. Secondly, since the total amount of posts and telecommunications is a comprehensive index reflecting the output of information infrastructure, so we use this comprehensive index (ln*post*) as a surrogate variable for the digital economy in regression estimation. Thirdly, due to the adverse influence of the international financial crisis, the export scale of many cities in China declined sharply in 2009, which was inconsistent with the changes in other years. Therefore, this paper deleted the sample data of 2009 and then made regression estimation on the benchmark model. The robustness test is shown in Table 3. It can be seen that the digital economy has a significant positive effect on the city export scale, and the results are relatively robust.

**Table 3:** Results of robustness test of the benchmark model.

Variable	Considering endogeneity	Replacing explanatory variable	Eliminating outliers
	ln*EX*	ln*EX*	ln*EX*
*Digit*	2.356**		1.348***
	(2.331)		(2.705)
ln*post*		0.090***	
		(2.819)	
*fdi*	0.039***	0.038***	0.033***
	(4.258)	(3.072)	(3.026)
*is*	-0.020***	-0.022***	-0.023***
	(-6.340)	(-2.989)	(-2.973)
*grp*	1.007***	1.023***	1.026***
	(8.050)	(3.128)	(3.008)
ln*hr*	0.636***	0.594***	0.623***
	(4.004)	(2.783)	(3.120)
ln*rd*	0.089	0.061	0.077
	(0.807)	(0.596)	(0.673)
Constant	11.727***	10.842***	11.625***
	(41.195)	(15.808)	(22.789)
City effect	Yes	Yes	Yes
Year effect	Yes	Yes	Yes
Observations	3990	4275	3990

Notes: 1. The t-statistic is in parenthesis.

***, **, and * indicate statistically significant at 1%, 5%, and 10%, respectively.

## Analysis of heterogeneity

### Heterogeneity in different city types

According to the results of the benchmark model, the digital economy does have a positive influence on the export scale of prefecture-level cities in China, but whether this influence varies by city characteristics? In this regard, the paper further classifies 285 prefecture-level cities to examine the heterogeneity of the effects of the digital economy on export at different characteristics.

**Fig 2:**
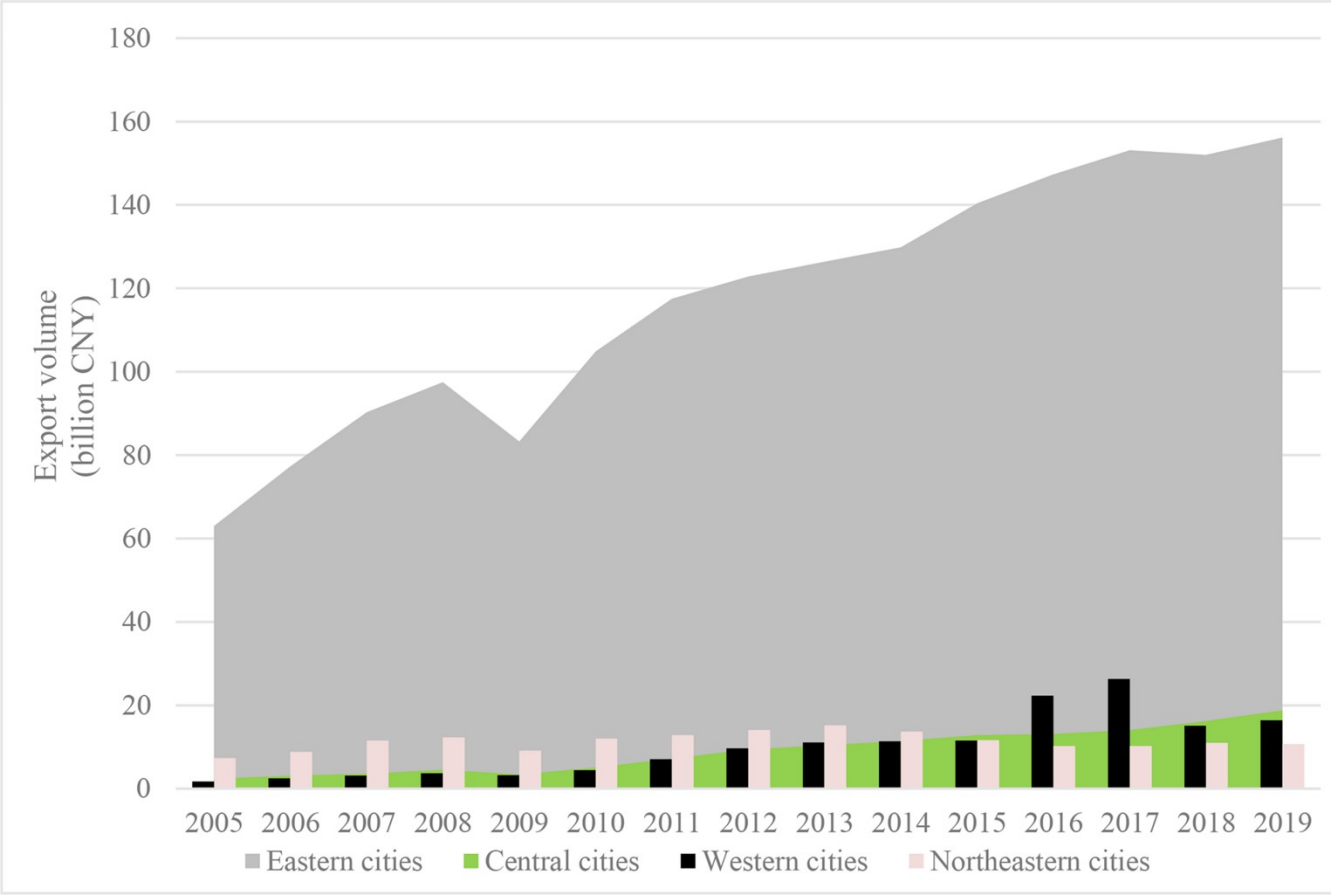
Average city exports at the different geographic locations.

**Fig 3:**
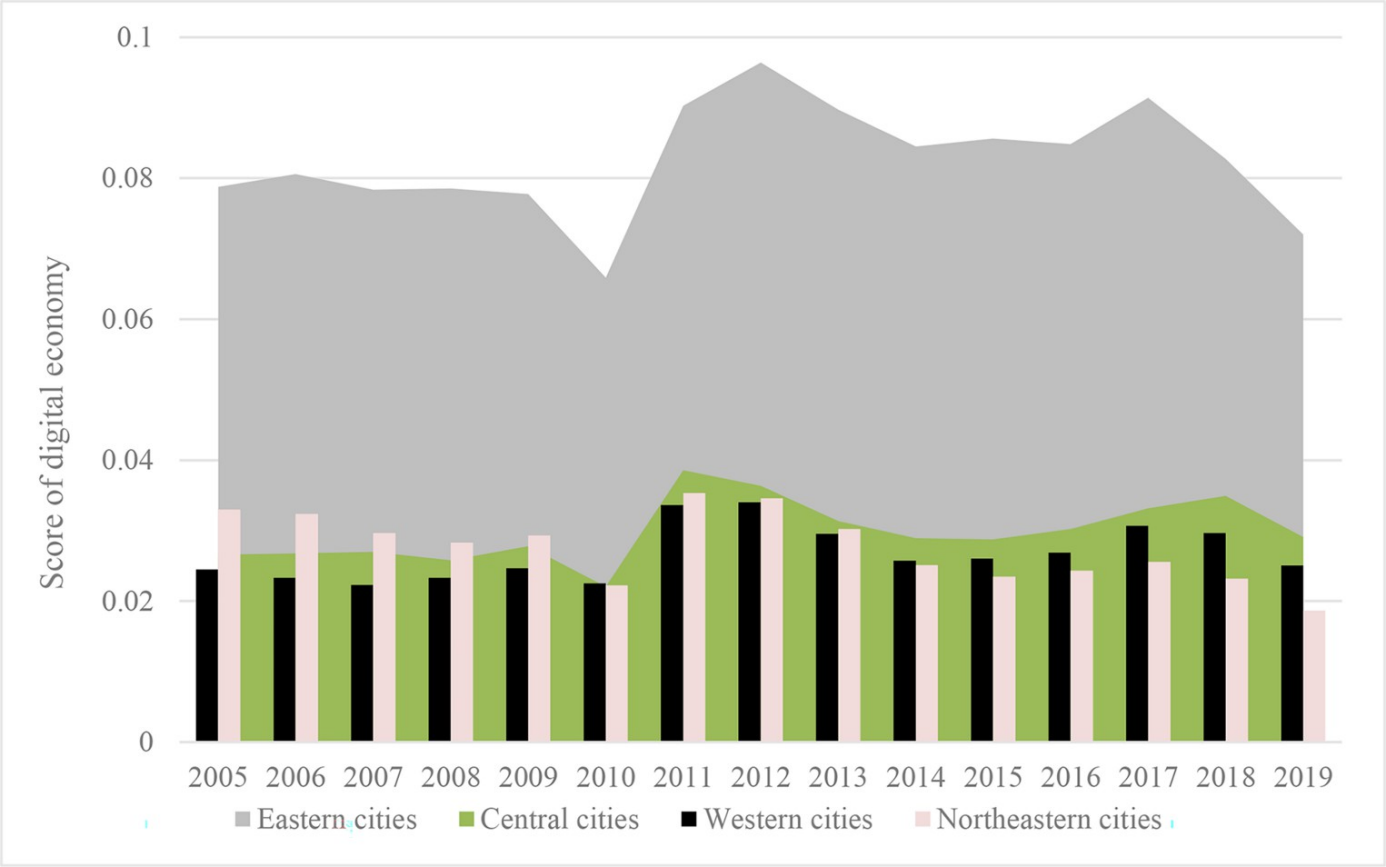
Digital economy development level at the different geographic locations.

Specifically, referring to the Division Method of East, West, Central, and Northeast Regions by the National Bureau of Statistics of China, we divide 285 prefecture-level cities into western cities, central cities, eastern cities, and northeastern cities according to their geographic locations. Geographical location will affect a city’s trade convenience and trade propensity: (1) By virtue of the geographical location near the port and the industrial advantages of the Yangtze River Delta and the Pearl River Delta, the export volume of the eastern region has always been far ahead of other regions. (2) Most of the central cities are located on the Yangtze River Golden Waterway, with numerous ports and well-developed shipping. For example, the throughput capacity of Wuhan Port, Jiujiang Port, and Ma’anshan Port has increased rapidly in recent years, providing certain conditions for export trade. (3) The western region is relatively closed geographically, influenced by topography and other factors, making infrastructure construction more difficult and not having the location advantage for trade exchanges. (4) The industrial structure of northeast China is dominated by traditional industries such as petrochemicals and iron and steel, with a relatively low share of emerging industries, which has resulted in insufficient economic growth momentum and relatively weak export competitiveness in recent years. Under different geographical locations, the city’s average export volume and digital economy development level are shown in Figs 2 and 3, respectively. Obviously, we can assume that the better the location of a city, the higher the city’s digital economy level and export scale.

Considering that the sample size of some types is too small, we divide all cities into two categories in regression, including eastern and central cities and western and northeastern cities. According to the sub-sample regression estimation results in Table 4, we find that the coefficients of *Digit* under different city types are significantly positive, which indicates that the digital economy is conducive to promoting the growth of export trade. However, by comparing the estimated results of columns (1) and (2) in Table 4, it suggests that the coefficient of variable *Digit* in column (2) is relatively larger; that is, the digital economy has a more noticeable effect on the export of cities in the western and northeastern regions than in the central and eastern regions.

**Table 4:** Results of heterogeneity test.

Variable	(1)	(2)	(3)	(4)
	Eastern and central cities	Western and northeastern cities	First- and second-tier cities	Third-tier and below cities
*Digit*	0.860*	4.675**	1.417**	5.312***
	(1.953)	(2.387)	(2.462)	(3.419)
*fdi*	0.042***	0.024**	0.041***	0.032**
	(2.770)	(2.278)	(7.681)	(2.153)
*is*	-0.024***	-0.018	-0.024***	-0.020**
	(-3.256)	(-1.537)	(-4.782)	(-2.258)
*grp*	0.496***	1.920***	0.040	1.644***
	(3.839)	(3.209)	(0.708)	(3.704)
ln*hr*	0.010	1.527***	0.036	0.918***
	(0.128)	(2.742)	(0.478)	(3.137)
ln*rd*	-0.147***	0.348	-0.261***	0.380***
	(-2.699)	(1.546)	(-2.248)	(3.337)
Constant	13.610***	8.898***	15.571***	10.353***
	(32.165)	(10.455)	(47.308)	(14.819)
City effect	Yes	Yes	Yes	Yes
Year effect	Yes	Yes	Yes	Yes
Observations	2505	1770	735	3540

Notes: The t-statistic in parenthesis.

***, **, and * indicate statistically significant at 1%, 5%, and 10%, respectively.

**Fig 4:**
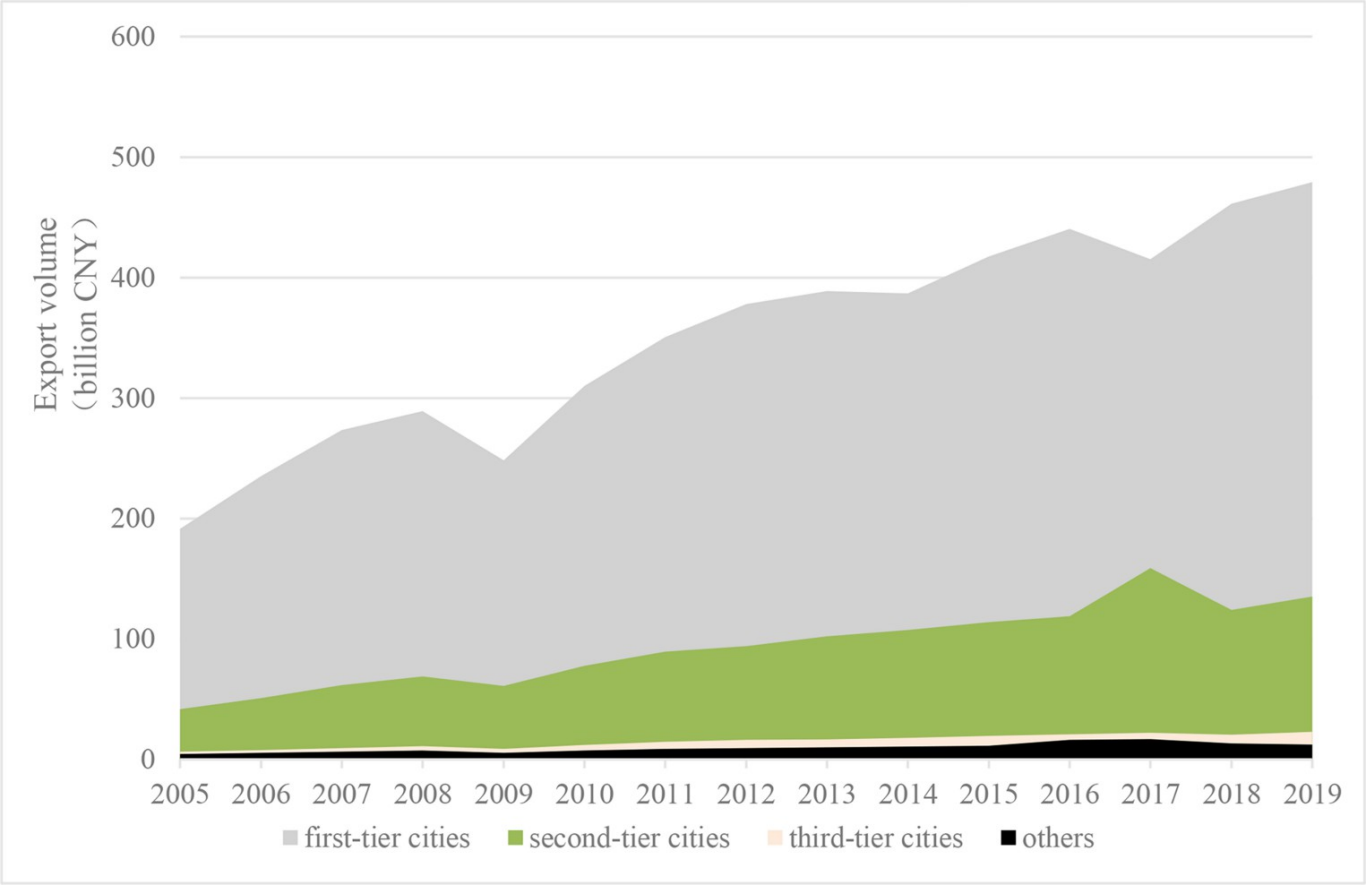
Average city exports at different development of the city economy.

**Fig 5:**
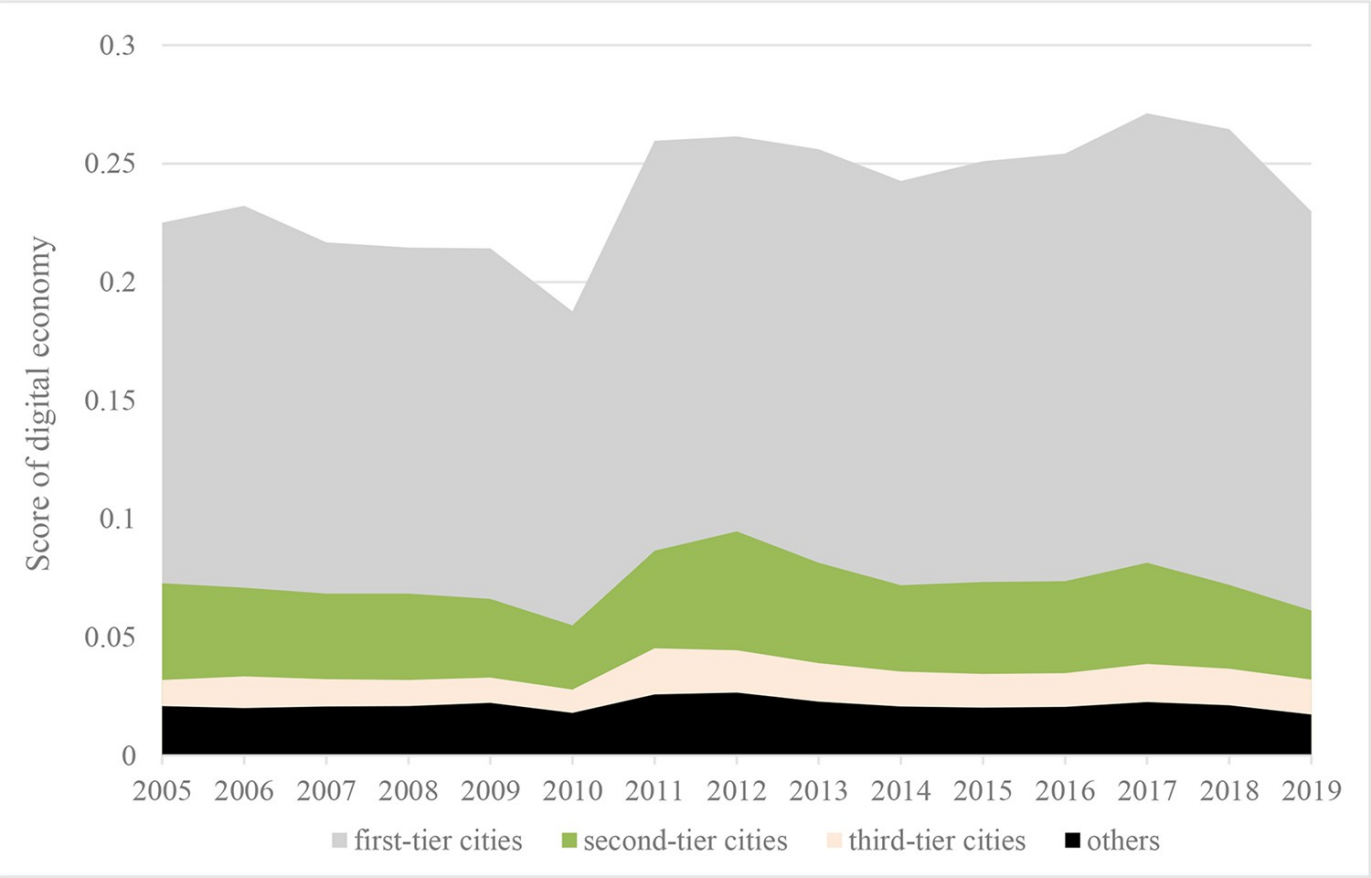
Digital economy development level at different development of the city economy.

Further, based on a comprehensive consideration of the city’s economic aggregate, political function, city size, and economic radiation, we divide the cities into first-tier cities, second-tier cities, third-tier cities, and others. The stronger developed the comprehensive economic strength, the better the economic environment, human resources, financial conditions, infrastructure, and so on, which lays a solid foundation for the export trade and digital economy of the city. As shown in Figs 4 and 5.

It is also considered that the sample size of some types is too small, we divide the sample into first- and second-tier cities, third-tier and below cities based. According to columns (3) and (4) in Table 4, we can conclude that the digital economy has a more significant effect on the export of third-tier and below cities compared to first-tier and second-tier cities.

Based on the analysis of the above two aspects, we consider that the digital economy plays a more significant role in promoting the export scale of cities with relatively small scales and relatively low levels of economic development.

### Robustness of heterogeneity

For the stage of city development, the previous section measured it mainly based on the city population size and the level of economic growth. Generally speaking, the export scale of cities with a larger population scale and a higher level of economic growth is also relatively larger. Hence the quantile regression model tests the robustness of the heterogeneity effect obtained by sub-sample regressions. Based on the results in Table 5, we can find that the estimated coefficients of *Digit* show a decreasing trend as export size gradually increases, and the coefficient is insignificant at the 90% quantile, which indicates that the boosting effect of the digital economy is no longer significant after the city export size reaches the 90% quantile. The quantile regression results above are consistent with the results of the sub-sample regression in heterogeneity, which can reflect the heterogeneity characteristics of the influence of the digital economy on export from different aspects. Thus Hypothesis 2 is confirmed.

**Table 5:** Results of quantile regression.

Variable	(1)	(2)	(3)	(4)	(5)
	10%	30%	50%	70%	90%
*Digit*	1.859**	1.570***	1.334***	1.122**	0.899
	(0.743)	(0.555)	(0.465)	(0.466)	(0.549)
*fdi*	0.032***	0.032***	0.032***	0.032***	0.032***
	(0.012)	(0.009)	(0.007)	(0.007)	(0.009)
*is*	-0.019***	-0.010***	-0.003	0.003	0.00***
	(0.004)	(0.003)	(0.003)	(0.003)	(0.003)
*grp*	2.801***	2.620***	2.473***	2.341***	2.202***
	(0.190)	(0.142)	(0.119)	(0.119)	(0.140)
ln*hr*	1.204***	1.131***	1.071***	1.017***	0.961***
	(0.261)	(0.195)	(0.163)	(0.163)	(0.193)
ln*rd*	0.855***	0.809***	0.772***	0.739***	0.704***
	(0.153)	(0.114)	(0.096)	(0.096)	(0.113)
Observations	4275	4275	4275	4275	4275

Notes: The standard error in parenthesis.

***, **, and * indicate statistically significant at 1%, 5%, and 10%, respectively.

## Analysis of influence mechanism: Mediation test

### Results of mediation test

After empirically verifying the promotion effect of the digital economy on exports and its heterogeneity, this paper further tests the specific influence mechanism. According to Eqs (1)–(3), we use the bias-corrected percentile Bootstrap method to test whether technological progress plays a mediating role in the digital economy affecting city export trade. From the estimation results in Table 6, in terms of direct effect, the coefficient is 5.374, which indicates that the impact of the digital economy on export trade is significant after controlling for the mediating variable of technological progress. In terms of the transmission mechanism of the mediating variable, the promotion effect of the digital economy on technological progress is notable, and technological progress significantly promotes the growth of export trade, so the product of the regression coefficients of the two is positive. That is, the indirect effect of technological progress on export trade is positive with a coefficient of 0.293, and the indirect effect does not contain 0 in the 95% confidence interval, indicating that the mediating effect of technological progress is established.

**Table 6:** Results of mediation test.

	Effect Path	Effect	Coefficient	95% conf. interval
**Bootstrap**	Digit→lnTFP→lnEX	Indirect	0.293*** (0.073)	[0.161,0.446]
	Digit→lnTFP→lnEX	Direct	5.374*** (0.713)	[4.201,7.017]
Mediating effect	**Significant**			

Notes: The standard error in parenthesis.

***, **, and * indicate statistically significant at 1%, 5%, and 10%, respectively.

### Robustness of mediation test

Similar to the robustness test of the benchmark model, we conducted a robustness test on the mediating effect from two aspects of eliminating outliers and replacing explanatory variables. The results of the bootstrap test are shown in Table 7, from which it is clear that technological progress still plays a significant mediating role in the promotion of export trade by the digital economy after removing the data from 2009 and using total postal business as a substitute variable for the digital economy. Therefore, Hypothesis 3 is confirmed.

**Table 7:** Results of the robustness of mediation test.

	**Effect Path**	**Effect**	**Coefficient**	**95% conf. interval**
**Eliminating outliers**	Digit→lnTFP→lnEX	Indirect	0.296*** (0.088)	[0.153,0.485]
	Digit→lnTFP→lnEX	Direct	3.471*** (0.668)	[2.052,4.741]
Mediating effect	**Significant**			
	**Effect Path**	**Effect**	**coefficient**	**95% conf. interval**
**Replacing explanatory variable**	lnpost→lnTFP→lnEX	Indirect	0.024*** (0.008)	[0.010,0.040]
	lnpost→lnTFP→lnEX	Direct	1.151*** (0.044)	[1.065,1.240]
Mediating effect	**Significant**			

Notes: The standard error in parenthesis.

***, **, and * indicate statistically significant at 1%, 5%, and 10%, respectively.

## Discussion

Analyzing the benchmark regression results, it shows that the digital economy significantly promotes city export trade, which is consistent with the research results of Lendle et al. [15], Goldfarb and Trefler [18], and Fan et al. [14]. They all concluded that digital technologies can reduce trade costs and expand the market boundaries of trade, thus the development of the digital economy is conducive to export trade.

In addition, the results of city heterogeneity reveal that the effect of the digital economy in promoting city export trade is influenced by city characteristics. For the relatively remote cities in the western and northeastern regions, as well as the third-tier and lower-tier cities with relatively backward economic development, the promotion effect of the digital economy is more pronounced. This is similar to the results of Farboodi and Veldkamp [26] and Clarke [27], but different from those of Pan et al. [25] who argue that cities with better location advantages and higher levels of economic development are more able to benefit from the development of the digital economy. One possible reason for this is that the western and northeastern regions are not advantageous in carrying out trade activities due to their geographical location and environment. The base of export trade is too small, so the impact of the digital economy enhancement on exports is more obvious. In relatively backward cities, due to the lack of factors of production, the economies of scale and scope generated by minor improvements in infrastructure are more obvious, so the marginal utility of the digital economy is greater.

What’s more, by analyzing the regression results of the mediating effect model, we find that technological progress plays a mediating role between the digital economy and export trade, and the mediating effect is 0.293. The digital economy speeds up the circulation of market information and production factors, which can improve the overall technical level and production efficiency [30]. And evidence shows that technological progress boosts export growth by improving export competitiveness [36].

## Conclusion

Based on the data of 285 prefecture-level cities in China from 2005 to 2019, this paper empirically analyzes the influence of the digital economy on city export trade and its heterogeneity. It also uses the mediating effect model to test the mediating role of technological progress to reveal the influence mechanism of the digital economy. The main conclusions are as follows: Firstly, the digital economy can promote the growth of export trade of prefecture-level cities in China; Secondly, due to the different stages of economic development in different cities, the promotion effect of the digital economy on city export scale is heterogeneous. Finally, technological progress plays a significant mediating role in promoting city export trade in the digital economy, and there is an influence mechanism of “digital economy-technological progress-city export trade”.

## Policy implications

In summary, it can obtain the following policy implications from this paper.

First, continue to increase investment in the construction of digital economy infrastructure to give full play to the role of the digital economy in promoting export trade. Infrastructure such as wireless base stations, communication networks, and relay stations are important hardware carriers for information transmission and exchange. Strengthening related infrastructure construction will be conducive to the growth of export trade in cities and the whole country. However, it should also be noted that the construction of digital economy infrastructure is a systematic project, which requires the organic combination of human, financial, and material resources and the effective unification of talents, strategies, and resources. In this regard, the government should increase infrastructure investment to promote the construction of fiber-optic networks and wireless networks. Meanwhile, it should strengthen policy support for related professionals to meet the demand for professional talents for city development.

Second, focus on the different development stages of different cities and tap the development potential of the digital economy. Digitalization has created a new gap between the rich and the poor, forming a “digital divide”. In response to the reality of e development of the digital economy in different regions of China, policymakers should focus on strengthening the digital construction power of the most backward areas of development. For example, to increase the investment in information network infrastructure in the most backward areas and to support and attract new service enterprises, digital technology talents, advanced technology, capital, and other quality production factors to gather in the backward areas through financial subsidies, to provide the material basis for the development of the digital economy. In addition, we also need to pay attention to the publicity and popularization of knowledge about the internet and digitalization in backward areas to reduce the effect of “not-in-my-yard”, which refers to the planning of economic projects or public facilities by government departments in which the whole society shares the benefits generated, but the nearby residents bear the negative externalities, so this plan is vehemently opposed and protested by the residents around the chosen site. It aims to ensure the smooth construction of digital infrastructure and fully tap the role of the digital economy in promoting city export trade.

Third, vigorously support the research and development of digital technologies and continue to boost the innovative growth of trade. We should attach importance to the R&D of key technologies in digital infrastructure construction and turn the list of “bottleneck” technologies that restrict the construction of digitalization into a list of scientific research tasks. The government needs to support enterprises, universities, and other innovation subjects to increase their independent research and development efforts to stimulate the growth potential of technological progress. In particular, it is necessary to closely follow the digital development strategy and the socio-economic development needs, scientifically distribute R&D investment in fields such as 5G, big data, and blockchain, and establish first-mover advantages in innovative trade.

## Limitation and future research directions

This paper still has the following limitations: In terms of the research object, we investigate the macro level but do not go deep into the micro level; In terms of the construction of the digital economy index system, the indicators chosen are not comprehensive enough because of the difficulty of obtaining complete data at the prefecture-level city level; In terms of the selection of mediating variables, technological progress only plays a partial mediating role in the influence of the digital economy on city export trade, then there may be other mediating variables, but this paper has not yet analyzed in this regard.

Future research can explore the relationship between the digital economy and export trade at the enterprise level. As a major player in export trade, the export capacity of enterprises directly affects the overall trade strength of China. At the same time, the construction of the digital economy index should include more aspects to ensure objectivity and fairness. In addition, when exploring the influence mechanism, mediating and moderating effects can be considered, and multiple influence mechanism variables can be selected for analysis.

## Supporting information

S1 Table The data of all variables. (ZIP)
